# Assessing Nigerian Butchers’ Knowledge and Perception of Good Hygiene and Storage Practices: A Cattle Slaughterhouse Case Analysis

**DOI:** 10.3390/foods10061165

**Published:** 2021-05-22

**Authors:** Charles Odilichukwu R. Okpala, Obichukwu Chisom Nwobi, Małgorzata Korzeniowska

**Affiliations:** 1 Department of Functional Food Products Development, Faculty of Biotechnology and Food Science, Wrocław University of Environmental and Life Sciences, 51-630 Wrocław, Poland; malgorzata.korzeniowska@upwr.edu.pl; 2 Department of Veterinary Public Health and Preventive Medicine, University of Nigeria Nsukka, 410001 Nsukka, Nigeria; obichukwu.nwobi@unn.edu.ng

**Keywords:** butchers, cattle industry, food safety, food hygiene, healthy workplace, slaughterhouse

## Abstract

In Nigeria, the National Agency for Food and Drug Administration and Control (NAFDAC) guides the inspection and production of beef meat and prescribes the good practices pertinent to beef products’ handling, processing, and packaging. Specifically, good hygiene practice (GHP) assures beef product safety and consumer protection, whereas good storage practice (GSP) assures the continuity of hygiene activities within the storage stages. Relevant literature about butchers’ knowledge and perception of good hygiene and storage practices within Nigeria slaughterhouses remains scant. This current study, therefore, assessed butchers’ knowledge and perception of good hygiene and storage practices through a cattle slaughterhouse case analysis. The selected cattle slaughterhouse serves the increasingly thriving Nsukka beef market in Enugu State, Nigeria. Content validation was utilised to authenticate the questionnaire items, which were administered face-to-face to the respondents (i.e., the butchers). The questionnaire included a total of 30 questions. The results showed that the butchers were male (Freq. = 100%, *n* = 50), acquired their knowledge informally (Freq. = 88%, *n* = 44), were largely with more than 5 years of slaughterhouse experience (Freq. = 82%, *n* = 41), and were strongly (*p* < 0.0001) familiar with good hygiene (Freq. = 96%, *n* = 48) and storage (Freq. = 98%, *n* = 49) practices. The butchers provided examples that demonstrated knowledge and perception aspects of GHP and GSP. The perception aspects of GHP and GSP were correlated more, compared to knowledge and knowledge versus perception. Very conscious of their knowledge and perception of good hygiene and storage practices, the butchers herein have to strive for continuous improvement in their slaughterhouse activities to assure beef quality and consumer safety.

## 1. Introduction

Globally, the demand for beef is continually on the rise, providing a highly desirable eating experience, even in developing countries [1]. In Africa, livestock production remains a very important aspect of the agro-ecological landscape [2]. Cattle are among the most important livestock species reared via traditional pastoral farming systems, particularly within northern Nigeria [3]. The cost of beef meat is less in the northern part compared to the southern part of Nigeria. Despite this, beef meat remains the major animal protein consumption resource [4] across households, communities, states, and the entire country. For the meat industry to thrive, there has to be a strategy that integrates the livestock value chain, consumers, and producers. Although Nigeria’s livestock industry appears not to be rapidly growing relative to the population that relies on it for beef, the number of grazing livestock as of 2010 was 108.6 million [5]. Notwithstanding meat processing largely dependent on livestock production [6,7], the beef market value creates employment and generates income for butchers. Equally, slaughtering and dressing cattle comes at a reasonable cost [4]. According to the Food and Agriculture Organisation (FAO) of the United Nations, meat products, either in fresh or frozen conditions, very rapidly become highly susceptible to microbial contamination as soon as they are exposed. It is exactly this situation that makes the hygiene of meat processing very relevant [8]. In addition, butchers at slaughterhouses in Nigeria, probably owing to their contact with a sizeable number of slaughtered cattle, could be exposed to zoonotic diseases [9], which makes relevant the need for optimum hygiene and storage practices.

In Nigeria, the Food Safety and Applied Nutrition (FSAN) Directorate under the National Agency for Food and Drug Administration and Control (NAFDAC) guides the inspection as well as the production at small–medium food enterprises and prescribes the minimum good hygiene practices (GHPs) pertinent during manufacturing, processing, and packaging, which are very applicable to cattle beef products. The fundamental aim is to assure both (meat) product safety and consumer protection. Further, beef production necessitates that technical skills, as well as equipment, be adequate and meet the regulated quality control standards [10]. Through the Federal Ministry of Health in Nigeria, the food safety management strategies provide the meat industry with prerequisite GHP regulatory guidelines [11]. Additionally, quality control mechanisms within the slaughterhouse environment, the butchers’ workspace in particular, must operate optimally to curb any form of bacterial/zoonotic contamination [12,13]. GHP essential measures, very relevant at each stage of the supply chain, appear strongly associated with food production, quality, and safety [14,15]. Good storage practice (GSP) on the other hand, and specific to the meat industry, considers incoming material storage to finished product storage. GSP ensures that there is continuity of hygiene activities in the meat-processing stage, e.g., after the first washing of beef, it is either frozen or refrigerated [16,17]. Effective beef storage is among the means of implementing good practice. It involves high discipline standards from personnel and documentation to storage environment/facilities [16].

**Figure 1:**
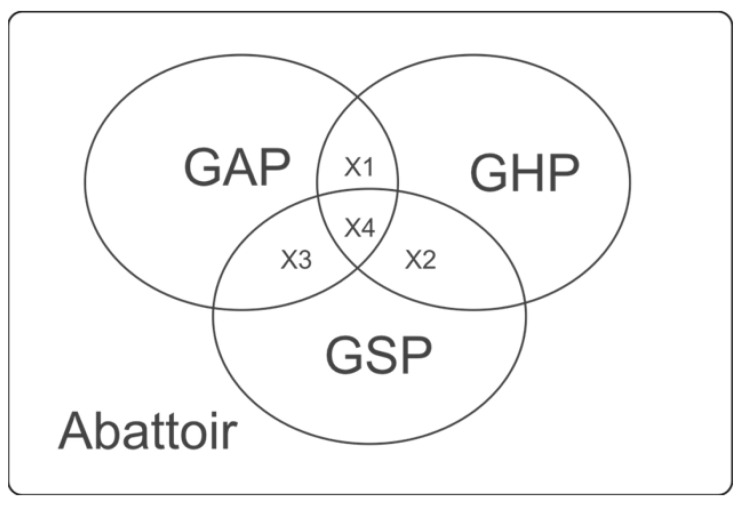
Schematic representation of the interaction of three key good practices applicable and relevant to a typical cattle abattoir/slaughterhouse in Nigeria. GAP = Good Agricultural Practice, which can involve the humane handling of cattle as well as pre-slaughter keeping of cattle at lairage; GHP = Good Hygiene Practice, which can involve slaughtering activity, as well as carcass splitting and inspection activities; GSP = Good Storage Practice, which can involve carcass storage and refrigeration; X1, X2, and X3 represent the interactive spaces of GAP × GHP, GHP × GSP, and GAP × GSP, respectively. In addition, X4 represents the interaction of GAP × GHP × GSP.

Described in codes of practice and largely designed by government bodies, good practices are known to involve quality assurance activities, which have to adhere to the control of food production and its associated processes [18]. Good practices form the crux of food quality and consumer protection, as well as within the agro-food industry. In addition to actualising the desired successes for food quality and consumer protection, the functioning of good practices ought to cut across all the production processes of agro-food products [19] and certainly to all the relevant facets of the meat industry. A schematic representation of the interaction of three key good practices applicable and relevant to a typical slaughterhouse in Nigeria is depicted in Figure 1. From cattle arriving in good condition (representing good agriculture practice (GAP)) to preparation for slaughter, the actual slaughter process, and the (immediate) carcass handling procedures after slaughter (representing GHP) to all aspects of carcass storage and sale procedures (representing GSP), there appears areas where these three clearly interact. Presumably, cattle rearers are largely involved with GAP, whereas GHP and GSP are of emphasis in the cattle slaughterhouse.

In contrast with developed countries, butchers in Nigeria largely operate under the administration and registration of the respective local government area (LGA) where the slaughterhouse is situated. In addition, the LGA mandates the butchers to work responsibly within their respective slaughterhouses, consistent with good hygiene and storage practices. What butchers know and perceive about good hygiene and storage practices would help the local, state, and federal governments, together with stakeholders, formulate problem-solving strategies for the cattle/meat industry. However, relevant literature about what butchers in Nigeria know and perceive of good hygiene and storage practices within the slaughterhouses where they work remains very scant. In this context, this current study aimed to assess butchers’ knowledge and perception of good hygiene and storage practices through a case analysis of a cattle slaughterhouse that serves the increasingly thriving Nsukka beef market in Enugu State, Nigeria.

## 2. Study Subjects and Methods

### 2.1. Schematic Overview of Current Study

**Figure 2:**
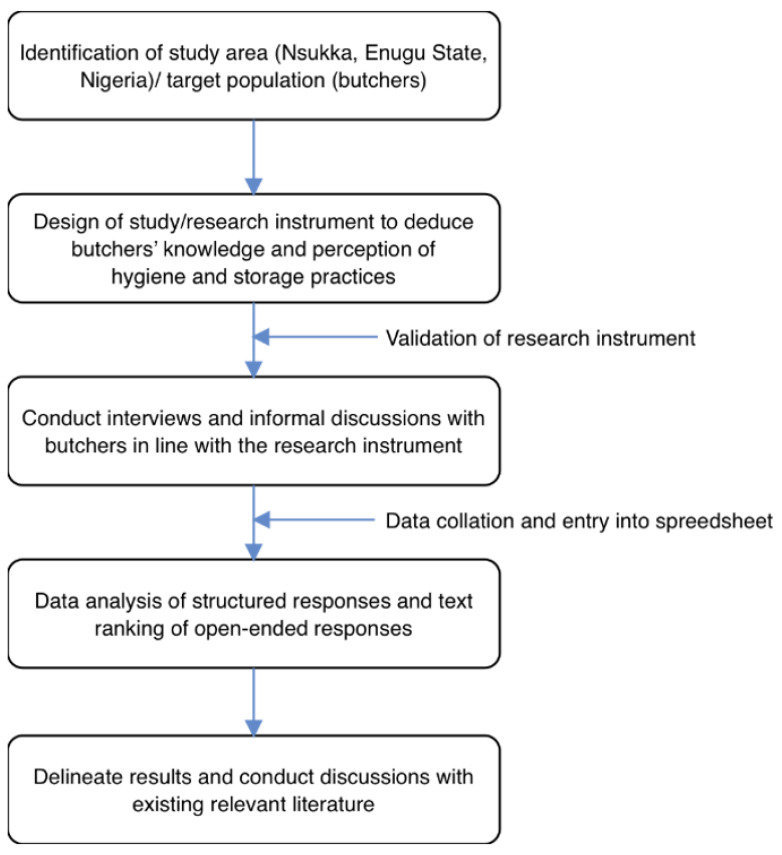
Schematic overview of current study, including the design of questions, conduct of interviews and informal discussions with butchers, data analysis, and delineating the results and discussing them using existing relevant literature.

The schematic overview of the current study, from the identification of the study area/target population and development of the research instrument to delineating the results and discussions using the existing body of knowledge, is shown in Figure 2. For emphasis, this current work was designed to assess Nigerian butchers’ knowledge and perception of good hygiene and storage practices via a case analysis of a cattle slaughterhouse. Essentially, this specific slaughterhouse was selected because of its important role, from receiving and slaughtering the cattle and processing and packaging the beef to supplying the beef to the Nsukka market (Enugu State, Nigeria).

### 2.2. Ethical Approval

Institutional ethics approval was not required for this study for the reason that it was strictly a questionnaire-based survey. However, the approval to use the research instrument for this survey was given by the slaughterhouse association. Additionally, this study adhered to the code of ethics of the World Medical Association Declaration of Helsinki [20]. Specifically, the informed consent was orally obtained from all butchers who participated in this study. In addition, the butchers’ participation at this study was voluntary.

### 2.3. Study Area and Target Population

The study area was Nsukka urban in Enugu State, Nigeria. Specifically, Nsukka urban is situated at latitude 6°45′ and 7° N and longitude 7°12.5′ and 7°36′ E. With a population increasing beyond 1.26 million, Nsukka is a well-known local government area (LGA) that situates an ever-growing metropolis/university community [21]. Butchers within the Nsukka slaughterhouse were the target population, who by experience, expertise, and delivery of services are typically representative of other slaughterhouses in terms of size, volume, as well as workforce, not only situated in various communities/LGAs around the state but also around the entire country.

### 2.4. Development of Research Instrument

The authors’ combined specialist experience/expertise and knowledge together with synthesised relevant literature helped in developing the research instrument, which followed a questionnaire approach. As highly recommended by Taherdoost [22], content validation was applied to ensure the (interview) questions were authentic and reliable. The validation process of the research instrument involved a specialist veterinarian together with a lead butcher, both with combined substantial years of cattle slaughter and slaughterhouse experience. During the content validation process, the questions of the research instrument were critically looked at and amended where deemed necessary, which strengthened the research instrument’s relevancy and representation to the targeted research construct/context [22]. The administration of the Butchers Association, represented by the lead butcher, participated in the validation process and subsequent approval of the research instrument (questionnaire). Once approval was obtained, the administration of the questionnaires proceeded.

### 2.5. Questionnaire Items, Slaughterhouse Workday, and Interview Process

The interview activity, based on questionnaire items, was composed of three sections: (a) demography and self-hygiene; (b) knowledge of good hygiene and storage practices; and (c) perception of good hygiene and storage practices in the slaughterhouse facility. The questionnaire included a total of 30 questions. Demography and self-hygiene had 6 questions, knowledge of good hygiene and storage practices had 11 questions, and perception of good hygiene and storage practices had 13 questions. A number of these questions required either a ‘yes’ or ‘no’ response, with a few others having other categorised responses. Importantly, a number of the questions were open ended, for example, asking ‘which’ and ‘why’. The purpose here was to allow the butchers to provide examples and thoughts where possible. This platform also gave some avenue for some informal discussions. Importantly, the number of questionnaire items were considered not too many. This is because too many items might deter the butchers’ participation. In general, the interviews were conducted during slaughterhouse visits and face-to-face with the interviewees, i.e., butchers. 

A total of 72 butchers were approached during the survey period. Out of these, questionnaires were administered to a total of 50 butchers, with no butcher participating more than once. This is because a few of them were very occupied at the time and therefore could not participate, whereas a few others were not open to answering the questionnaire. Specifically, the typical slaughterhouse workday involves, among other activities, the cattle being presented for slaughter. The butchers, in addition to humanely handling the slaughtering of cattle and the processing of the carcass, remain actively involved up to the point of sale of the beef and its associated parts. A butcher, with the help of one or more assistants (junior butchers), handles both the slaughtering and the processing of the cattle. The method of cattle slaughter is performed using the transverse cut method. Thereafter, the animal is immobilised and placed in such a way that the head is well restrained before one stroke of a cut across the throat is made with a very sharp knife severing the trachea, oesophagus, and blood vessels. This traditional method allows the animal to be well bled. Besides this, other butchers not directly involved in the actual slaughter activity of a given cattle would be engaged in other slaughterhouse duties, from cleaning the slaughterhouse surroundings to preparing the slaughter slab and its vicinity.

The interview process was conducted by a veterinarian, supported by a trainee veterinarian. The veterinarian is familiar with the slaughterhouse and its personnel. The questionnaire items were administered in such a way to encourage participation. Sampling the butcher to interview was non-random but without any repetition, as carried out by the interviewer. The recruitment process was such that the interviewer approached butchers who were less occupied and engaged with them individually. The open questions provided the butchers the opportunity to freely express themselves, as they shared their thoughts. The interview process was such that the questions were posed to the butchers, and their responses were written down by the interviewer (veterinarian). Through this approach, a relaxed atmosphere was assured, and the interview time was optimised with least-to-no pressure created on the butchers. Given the relaxed atmosphere the interviewer created, the butchers were relaxed, which encouraged and facilitated them to come up with examples where they deemed appropriate.

Each interview started primarily by briefly relaying the objective of the study to the butcher. When this step was achieved, the next step was to conduct the interview by administering the items in the questionnaire to the butcher. Some of the butchers were interviewed while they were working, whereas some had already accomplished their daily tasks prior to the time the interview(s) commenced. In both scenarios, the butchers were able to speak freely and respond to the questions posed to them. The interview process was such that it assured their anonymity and encouraged proactive participation as well as a willingness to provide information in a non-biased, as well as an objective, manner.

To ease the interviewees’ understanding of questions especially when (understanding) difficulties arose, the use of the vernacular was applied without any change in both the content and context of the questionnaire items. Essentially, the interview time varied as it was dependent on both the availability and convenience of the interviewees (butchers). In the situation where the interviews could not be accomplished on that day, another time agreed by both parties, i.e., the interviewer and interviewees, was scheduled.

### 2.6. Statistical Analysis

All data were subject to the Anderson–Darling normality test in which the data fulfilled the assumptions of non-parametric distribution. Therefore, the Kruskal–Wallis analysis of variance (ANOVA) test was applied to establish whether any statistical difference existed between response groups/variables. The results of the analysed data are presented in frequencies, percentages, H-adjusted values, and p-values. Where the correlation was required, Spearman’s test was applied; both the correlation coefficient (r) and probability (p) values are reported. The level of statistical significance was set at *p* < 0.05. Minitab Express software (version 1.5.3, Minitab Ltd., Coventry, UK) was used to carry out the statistical analysis. Furthermore, the open-ended responses were then categorised using the word-based technique of Ryan and Bennard [23] with slight modifications. The actual texts, based on the butchers’ responses, were sorted in order to arrive at a specific statement/theme, after which the frequency of occurrence was tallied, ranked, and, thereafter, reported in percentages.

## 3. Results and Discussion

### 3.1. Demographic and Self-Hygiene of Butchers

The butchers’ demographic and self-hygiene statistics, as sampled in the slaughterhouse, are shown in Table 1. At the studied slaughterhouse, the butchers were male (*p* < 0.0001) (Freq. = 100%, *n* = 50), with more of the butchers having primary (Freq. = 50%, *n* = 25) compared to secondary (Freq. = 28%, *n* = 14) education. The male control at the slaughterhouse of this study is consistent with findings reported elsewhere [24,25,26,27,28,29]. The self-hygiene was assessed based on covering the hair, wearing of an apron, application, and frequency of cleaning the work area. Butchers who covered their hair and wore an apron did not significantly differ (*p* > 0.05) from those who did not. Butchers who applied only water (Freq. = 66%, *n* = 33) compared to water and soap (Freq. = 32%, *n* = 16) to clean the work area/space of the beef slaughter activity differed significantly (*p* < 0.0001), similar to the cleaning frequency of before and after (Freq. = 88%, *n* = 44) compared to now and then (Freq. = 10%, *n* = 5) (Table 1). Notwithstanding their inconsistencies in the use of the apron, the butchers appeared undeterred in their consciousness of their self-hygiene as well as in the frequency of cleaning their work area. Essentially, the butchers’ demographic and self-hygiene statistics are relevant because they reveal some very key fundamentals, such as the butchers’ level of education, how the butchers appear at the slaughterhouse workday specific to the context of hygiene, how they keep their slaughterhouse work area tidy, and how frequently they perform cleaning activities.

### 3.2. Butchers’ Knowledge of Good Hygiene and Storage Practices

Knowledge is said to accumulate through learning processes, which may come either by formal or informal education, together with personal experience as well as experiential sharing [30]. The statistics of butchers’ knowledge of good hygiene and storage practices are shown in Table 2. Butchers were strongly (*p* < 0.0001) familiar with good hygiene (Freq. = 96%, *n* = 48) and storage (Freq. = 98%, *n* = 49) practices. Largely, they had acquired the knowledge informally (Freq. = 88%, *n* = 44) and possessed >5 years of experience in the beef-processing sector (Freq. = 82%, *n* = 41). Butchers acquiring their knowledge of good hygiene and storage practices informally demonstrates the relevance of their apprenticeship style of (good hygiene and storage practice) training. Indeed, such an apprenticeship style, typically in an in-house setting, can still be considered as an essential professional package, wherein experiential knowledge and practice is transferred from a certified butcher to a learner/trainee during the slaughterhouse workday activities and remains continuous over a period of time. Moreover, the butchers herein provided some examples of good hygiene practices employed in the slaughterhouse, which ranked as follows: use of clean water and (antiseptic) soap to wash hands (Freq. = 54%, *n* = 27) >use of disinfectant, clean water, and brush to scrub workspace floor (Freq. = 42%, *n* = 21) >use of clean water to wash the fresh meat (Freq. = 34%, *n* = 17) >regular washing of knives, wearing an apron, and washing of other work instruments (Freq. = 24%, *n* = 12) >regular washing of tables/slabs where the meat is split and prepared for market (Freq. = 22%, *n* = 11) >covering fresh meat (from flies) after washing and during refrigeration and transportation (Freq. = 10%, *n* = 5) >sweeping out debris/dirt off slaughter areas and burning the debris/dirt (Freq. = 4%, *n* = 2).

From the conducted interviews herein, we opine that the butchers certainly have the concept of hygiene in their minds, and do think about it, despite the challenges they are confronted with during their slaughterhouse activities. Obviously, the knowledge of hygiene in butchers grows and eventually matures through their work experience over time. Additionally, the degree of hygiene knowledge would differ from butcher to butcher, even within the same slaughterhouse workplace. Probably in some of the more experienced butchers, the degree of hygiene knowledge exemplified during slaughterhouse activities would be higher than the less experienced ones. Despite the routine nature of the butchers’ work every day, there would likely be something new to learn, now and then. According to the World Health Organization (WHO) of the United Nations, the simple act of washing hands with soap and water reduces the incidence of diarrhoea caused by foodborne pathogens by up to 35% [30]. Most certainly, these routine cattle slaughter procedures and their associated activities, carried out over a period of time, help in consolidating butchers’ understanding of good hygiene practices in the slaughterhouse. The high perishable nature of meat makes the butchers’ knowledge level, particularly with respect to hygiene within the meat industry, very essential to ensure consumer health and safety [31].

Further, the butchers provided some examples of good storage practices obtained within the slaughterhouse, which ranked as follows: use of cold room (Freq. = 56%, *n* = 28) > use of (private) refrigerator/freezer (Freq. = 36%, *n* = 18), as shown in Table 2. Significantly fewer butchers (*p* < 0.0001) indicated prior hygiene and storage knowledge/experience before engaging in the slaughterhouse (Freq. = 20%, *n* = 10) and were able to name a foodborne pathogen associated with beef (Freq. = 8%, *n* = 4). The few butchers able to name a foodborne pathogen associated with beef could only provide the example of *Salmonella* (Freq. = 8%, *n* = 4). Despite this, significantly more butchers (*p* < 0.0001), also shown in Table 2, indicated knowing the importance of hand sanitisation and how to use the storage facilities in the slaughterhouse (Freq. = 96%, *n* = 48). Essentially, the inability of butchers to name a foodborne pathogen might not necessarily deter their commitment and diligence to duty or their capacity to connect effectively with good hygiene and storage practices. Moreover, the ranking of butchers’ responses to examples with respect to the knowledge aspects may well depict the differences in (their) emphasis on good hygiene and storage practices. Based on these knowledge aspects, the butchers have a responsibility to continually improve on the slaughterhouse services they render to the public.

### 3.3. Butchers’ Perception of Good Hygiene and Storage Practices

The statistics of butchers’ perception of good hygiene and storage practices are shown in Table 3. Butchers significantly (*p* < 0.0001) perceived both good hygiene and storage practices as very important (Freq. = 72%, *n* = 36). Despite this, a fair number, respectively, perceived the hygiene and storage level of the slaughterhouse facility as ‘not so high’ (Freq. = 48%, *n* = 24) and ‘very high’ (Freq. = 58%, *n* = 29). The response of ‘not so high’ might have arisen from butchers’ experience regarding hygiene challenges encountered in the slaughterhouse. When asked why good hygiene storage practices were important, butchers openly responded: ‘to prevent meat contamination/spoilage and foodborne disease spread’ (Freq. = 42%, *n* = 21) of equal response with ‘to maintain clean/disease-free beef that ensures public health and consumer safety’ (Freq. = 42%, *n* = 21), with ‘to promote butchers’ self-hygiene’ (Freq. = 6%, *n* = 3) as the least mentioned. In the beef industry/production, butchers remain among the critical stakeholders. This is because of the linkage butchers provide between potential zoonotic disease and the meat-processing chain, particularly at slaughterhouses [13].

The majority of butchers significantly agreed (*p* < 0.0001) that aspects of the hygiene and storage facilities of the slaughterhouse required improvement (Freq. = 90%, *n* = 45) and that the local, state, as well as federal governments had a role to play to enhance the slaughterhouse hygiene and storage facilities/practices (Freq. = 84%, *n* = 42) (Table 3). Indeed, inadequate storage practices (as well as facilities) are among the key challenges that confront the meat industry [1,3,6,16]. Moreover, the butchers herein also provided examples of the slaughterhouse hygiene/storage aspects that needed improvement, which ranked as follows: improving the water supply (Freq. = 28%, *n* = 14) > improving the conditions of the slaughter slab and vicinity (Freq. = 22%, *n* = 11) > improving the (waste) drainage system (Freq. = 16%, *n* = 8) > improving the electricity supply (Freq. = 14%, *n* = 7) > improving the refrigeration cold-room/freezing facilities (Freq. = 10%, *n* = 5) > provision of (more) beef-processing rooms (Freq. = 8%, *n* = 4). When asked which of either hygiene or storage practices presented a greater challenge, more butchers indicated ‘hygiene’ (Freq. = 58%, *n* = 29) over ‘storage’ (Freq. = 16%, *n* = 8). Clearly, within the slaughterhouse, the butchers encounter more challenges of hygiene compared with those of storage. When asked which among the local, state, and federal governments had a role to play to enhance the slaughterhouse hygiene and storage facilities/practices, a reasonable proportion of butchers indicated ‘local’ (Freq. = 74%, *n* = 37), much less the ‘state’ (Freq. = 8%, *n* = 4), with no mention of the ‘federal’ government (Freq. = 0%, *n* = 0).

With regard to government regulation, there was no statistical difference (*p* > 0.05) between those who believed and did not believe that the government regulation protects the slaughterhouse good hygiene and storage practices. In addition, there was no statistical difference (*p* > 0.05) between those who believed and did not believe that the government regulation helps to sustain the (slaughterhouse) facilities. Further, when asked about how government regulation helps to sustain the slaughterhouse facilities, the butchers’ open responses ranked as follows: monthly participation in the environmental clean-up of the vicinity (Freq. = 32%, *n* = 16) > some regulatory hygiene control of slaughtered beef production (Freq. = 12%, *n* = 6) > regulatory standard of butchers’ conduct (by LGA and Butchers Association) (Freq. = 10%, *n* = 5). There is a high chance that butchers would more likely associate with the local government and its own local association and cooperate effectively with the instituted (slaughterhouse) regulation. There is the need, therefore, for the government to rise up to their expected role in the slaughterhouse, particularly to increase hygiene levels, in order to enhance the butchers’ protection and safety. In addition, the butchers consider good hygiene and storage practices as an obligation as well as responsibility for the public good.

### 3.4. Correlation Outcomes of Knowledge and Perception Aspects

The correlation analysis between knowledge and perception aspects was conducted with respect to butchers’ responses to good hygiene and storage practices. Specifically, the correlation coefficients, respectively, obtained between highly responded butchers’ knowledge, perception, and knowledge and perception aspects of good hygiene and storage practices are shown in Table 4, Table 5 and Table 6. For the knowledge aspects, there were positive correlations between the familiarity of good storage practices and having more than 5 years of work experience in the beef-processing sector (*r* = 0.3049, *p* = 0.0313) as well as knowing the importance of hand washing and how to use the slaughterhouse storage facilities (*r* = 1.0000, *p* < 0.0001) (Table 4). The meat safety knowledge of butchers increased with their years of professional work experience [30]. On the other hand, the perception of good hygiene and storage practices being very important correlated positively with the perception of the hygiene level of the slaughterhouse being not so high (*r* = 0.5100, *p* = 0.0002), the (slaughterhouse) storage level being very high (*r* = 0.6426, *p* < 0.0001), the belief that the local, state, and/or federal governments had a role to play to enhance the (slaughterhouse) hygiene and storage facilities/practices (*r* = 0.4569, *p* = 0.0009), the belief that the government regulation protects the (slaughterhouse) good hygiene and storage practices (*r* = 0.5599, *p* < 0.0001), and the belief that the government regulation can help sustain the (slaughterhouse) hygiene and storage facilities (*r* = 0.5345, *p* < 0.0001) (Table 5).

The perception of the hygiene level of the slaughterhouse being ‘not so high’ correlated positively with the (slaughterhouse) storage level being ‘very high’ (*r* = 0.5743, *p* < 0.0001), the agreement that some aspects of hygiene and storage facilities needed improvement (*r* = 0.3203, *p* = 0.0234), the belief that the local, state, and/or federal governments had a role to play in enhancing the (slaughterhouse) hygiene and storage facilities/practices (*r* = 0.4193, *p* = 0.0024), the belief that the government regulation protects the (slaughterhouse) good hygiene and storage practices (*r* = 0.8430, *p* < 0.0001), and the belief that the government regulation could help sustain the (slaughterhouse) hygiene and storage facilities (*r* = 0.8006, *p* < 0.0001). The perception of the slaughterhouse storage level being ‘very high’ correlated positively with the belief that the government regulation protects (slaughterhouse) good hygiene and storage practices (*r* = 0.7235, *p* < 0.0001) and the belief that the government regulation could help to sustain (slaughterhouse) hygiene and storage facilities (*r* = 0.6889, *p* < 0.0001) (Table 5).

The agreement that some aspects of the hygiene and storage facilities needed improvement correlated positively with the belief that local, state, and/or federal governments had a role to play to enhance (slaughterhouse) hygiene and storage facilities/practices (*r* = 0.4001, *p* = 0.0040), the belief that the government regulation protects (slaughterhouse) good hygiene and storage practices (*r* = 0.3469, *p* = 0.0136), and the belief that the government regulation helps to sustain (slaughterhouse) hygiene and storage facilities (*r* = 0.3333, *p* = 0.0180). The belief that local, state, and/or federal governments had a role to play to enhance (slaughterhouse) hygiene and storage facilities/practices correlated positively with the belief that the government regulation protects (slaughterhouse) good hygiene and storage practices (*r* = 0.3451, *p* = 0.0141) and the belief that the government regulation helps to sustain (slaughterhouse) hygiene and storage facilities (*r* = 0.3273, *p* = 0.0203). The belief that the government regulation protects (slaughterhouse) good hygiene and storage practices correlated positively with the belief that the government regulation helps to sustain (slaughterhouse) hygiene and storage facilities (*r* = 0.9608, *p* < 0.0001) (Table 5). Clearly, the above-mentioned correlations suggest that butchers’ knowledge and perception of good hygiene and storage aspects can interact when considered in the practice point of view. In addition, the butchers herein to some degree appear very mindful of their knowledge and perception of good hygiene and storage practices within the slaughterhouse.

For the knowledge versus perception aspects, both negative and positive significant correlations were obtained. The possession of more than five years of work experience in the slaughterhouse correlated negatively with the perception of good hygiene and storage practices being ‘very important’ (*r* = −0.2922, *p* = 0.0395), the perception of the (slaughterhouse) hygiene level being ‘not so high’ (*r* = −0.4877, *p* = 0.0003), the perception of the (slaughterhouse) storage level being ‘very high’ (*r* = −0.3987, *p* = 0.0041), the belief that the government regulation protects (slaughterhouse) good hygiene and storage practices (*r* = −0.4501, *p* = 0.0010), and the belief that the government regulation helps to sustain (slaughterhouse) hygiene and storage facilities (*r* = −0.3644, *p* = 0.0093). On the other hand, the perception of good hygiene and storage practices being ‘very important’ correlated positively with knowing the importance of hand washing (*r* = 0.3273, *p* = 0.0203) as well as knowing how to use the slaughterhouse storage facilities (*r* = 0.3273, *p* = 0.0203) (Table 6). The negative correlation suggests that the greater the number of years of work experience at the slaughterhouse may not in every respect reflect the degree to which the butchers perceive the following: (a) how they see their good hygiene and storage practices in general; (b) how they see the (slaughterhouse) hygiene and storage levels; (c) how they see the capacity of government regulation to protect (slaughterhouse) good hygiene and storage practices; and (d) how they see the government regulation to help sustain (slaughterhouse) hygiene and storage facilities. Overall, the perception of good hygiene and storage practices appears to be correlated more, compared to knowledge and knowledge versus perception aspects. This might suggest that the butchers are able to reveal their knowledge of good hygiene and storage practices via perception. This is probably by the internalisation of the information gathered from daily routine activities that directly involve good hygiene and storage practices. Noteworthily, it might be neither too difficult nor take too much time for the butchers to implement the connection between the actual and theoretical knowledge of good hygiene and storage practices. The actual aspect, in this case, specifically refers to what the butchers were able to reveal about their knowledge of good hygiene and storage practices. The theoretical aspect, in this case, cumulates all scientific literature within the body of knowledge specific to good hygiene and storage practices.

## 4. Limitations

This current work involved a total number of 50 butchers within a specific slaughterhouse. This might be considered a limitation to this study because more butchers involving a number of slaughterhouses would most likely increase the study’s representativeness and robustness. Additionally, the nature of the sampling, which was non-random, could also be perceived as a limitation. The non-random sampling approach was selected given that butchers in only one slaughterhouse were being studied. Random sampling will be more applicable where a number of slaughterhouses are to be investigated. Even though we believe that the non-random sampling used in this study would not necessarily increase the bias, given that each butcher will share their own particular work experience, random sampling would definitely play a role to further reduce the bias. A perceived limitation could also be the ‘yes’ or ‘no’ answers given by the butchers in response to some specific questions, which might not necessarily reveal the truth. Additionally, the attributes of knowledge and perception studied herein might be perceived as somewhat preliminary, which could be perceived as another limitation. However, we believe the current study lays a foundation for more robust studies in the future. 

## 5. Conclusions

The butchers at the slaughterhouse were male, acquired their knowledge informally, and had, largely, >5 years of slaughterhouse experience. The majority of the butchers considered both good hygiene and storage practices as an obligation and responsibility for the public good. They equally agreed that there were aspects of the hygiene and storage facilities at the slaughterhouse that required improvement. They were able to provide examples that exemplified their knowledge and perception aspects of good hygiene and storage practices as employed within the slaughterhouse activities. Correlation coefficients were obtained between the highly responded knowledge, perception, and knowledge and perception aspects of good hygiene and storage practices. The perception aspects correlated a lot more, compared to knowledge as well as knowledge versus perception.

Very conscious of their knowledge and perception of good hygiene and storage practices, the butchers herein have to continually improve the slaughterhouse services to ensure beef quality and consumer safety. There is a need for the local, state, and federal governments to get more involved, in particular, to enhance the butchers’ hygiene and storage practices, as well as to improve the slaughterhouse facilities. In this context, this work has made an attempt to address an aspect of Nigerian butchers’ occupation that directly leads to enhancing livestock welfare and product health and safety. The reasons for assessing butchers’ knowledge and perception with respect to hygiene include finding out areas where they require support and understanding the general level of their hygiene knowledge, as well as how they perceive hygiene in the context of their slaughterhouse activities. Through this understanding, it would be possible to decipher the areas where butchers’ hygiene challenges emanate from and where the local, state, and federal governments, together with stakeholders, could step in in order to serve as a support system to help formulate problem-solving strategies for the cattle/meat industry. 

Overall, this current study lays the foundation for a call for future government-funded nationwide campaigns in Nigeria, which would help enhance the meat industry regulatory standards specific to good hygiene and storage competencies and the status of butchers and slaughterhouses. As many of the butchers had more than five years of slaughterhouse experience, future work could aim to deduce how the frequent usage of various cleaning facilities and procedures associates with their length/years of work experience. Another future study could be to identify the risk factors for poor hygiene or the ‘positive factors’ for good hygiene and corresponding responses to the other explanatory variables, and this can be achieved using logistic regression analysis. Another direction of future work should be to look at the function and role of HACCP in a typical slaughterhouse, particularly in the Nigerian context.

## Figures and Tables

**Table 1:** Butchers’ demographic and self-hygiene statistics, as sampled in the slaughterhouse.

Item	Sub-Item	Frequency % (n = 50)	H-adj.	*p*-Value
Gender	Male	100% (*n* = 50)	99.00	<0.0001
	Female	0% (*n* = 0)		
Education status	Primary	50% (*n* = 25)	13.73	<0.0010
	Secondary	28% (*n* = 14)		
	Tertiary	16% (*n* = 8)		
	Unknown	6% (*n* = 3)		
Hair of butcher	Covered	44% (*n* = 22)	-	>0.05
	Uncovered	44% (*n* = 22)		
	Unknown	12% (*n* = 6)		
Wearing an apron	Used	48% (*n* = 24)	-	>0.05
	Not used	42% (*n* = 21)		
	Unknown	10% (*n* = 5)		
Application of cleaning the work area	Water	66% (*n* = 33)	45.83	<0.0001
	Water+soap	32% (*n* = 16)		
	Water+soap+disinfectant	2% (*n* = 1)		
Frequency of cleaning the work area	Before and after each activity	88% (*n* = 44)	60.26	<0.0001
	Now and then	10% (*n* = 5)		
	Unknown	2% (*n* = 1)		

**Table 2:** Statistics of butchers’ responses regarding their knowledge of good hygiene and storage practices.

No.	Question	Response	Frequency % (*n* = 50)	H-adj	*p*-Value
1	Are you familiar with good hygiene practices?	Yes	96% (*n* = 48)	83.79	<0.0001
		No	4% (*n* = 2)		
2	Are you familiar with good storage practices?	Yes	98% (*n* = 49)	95.12	<0.0001
		No	0% (*n* = 0)		
3	If yes to 1/2, how did you acquire the hygiene and or storage practice knowledge?	Formal	6% (*n* = 3)	66.81	<0.0001
		Informal	88% (*n* = 44)		
4	Years of experience in the beef-processing sector	<1 year	0% (*n* = 0)	87.83	<0.0001
		1–5 years	14% (*n* = 7)		
		>5 years	82% (*n* = 41)		
5	Did you have prior hygiene/storage knowledge/experience before engaging in the slaughterhouse?	Yes	20% (*n* = 10)	31.10	<0.0001
		No	76% (*n* = 38)		
6	Do you know any foodborne pathogen associated with beef?	Yes	8% (*n* = 4)	66.59	<0.0001
		No	90% (*n* = 45)		
7	Do you know the importance of hand washing?	Yes	96% (*n* = 48)	91.38	<0.0001
		No	0% (*n* = 0)		
8	Do you know how to use the storage facilities in the slaughterhouse?	Yes	96% (*n* = 48)	91.38	<0.0001
		No	0% (*n* = 0)		

**Table 3:** Statistics of butchers’ responses regarding their perception of good hygiene and storage practices.

No.	Question	Response	Frequency (*n* = 50)	H-adj	*p*-Value
1	As a butcher, how do you perceive good hygiene/storage practice, in general?	Very important	72% (*n* = 36)	58.88	<0.0001
		Important	28% (*n* = 14)		
		Not sure	0% (*n* = 0)		
2	How do you perceive the hygiene level at the slaughterhouse facility?	Very high	12% (*n* = 6)	37.04	<0.0001
		High	38% (*n* = 19)		
		Not so high	48% (*n* = 24)		
		Not sure	2% (*n* = 1)		
3	How do you perceive the storage level at the slaughterhouse facility?	Very high	58% (*n* = 29)	61.66	<0.0001
		High	38% (*n* = 19)		
		Not so high	4% (*n* = 2)		
		Not sure	0% (*n* = 0)		
4	Are there some aspects of hygiene and storage at the slaughterhouse you believe require improvement?	Yes	90% (*n* = 45)	63.66	<0.0001
		No	10% (*n* = 5)		
5	Do you believe the local, state, and federal governments have a role to play to enhance the slaughterhouse hygiene and storage facilities/practices?	Yes	84% (*n* = 42)	45.78	<0.0001
		No	16% (*n* = 8)		
6	Do you believe the government regulation protects slaughterhouse good hygiene and storage practices?	Yes	52% (*n* = 26)	-	>0.05
		No	48% (*n* = 24)		
7	Do you believe the above (6) has helped to sustain slaughterhouse hygiene and storage facilities?	Yes	52% (*n* = 26)	-	>0.05
		No	48% (*n* = 24)		

**Table 4:** Correlation coefficients obtained between highly responded knowledge aspects of good hygiene and storage practices of butchers.

Variable	A1	A2	A3	A4	A5	A6	A7
A2	−0.0292 ^1^ 0.8407 ^2^						
A3	0.2387 0.0950	−0.0528 0.7160					
A4	−0.0956 0.5088	0.3049 0.0313 *	−0.1730 0.2295				
A5	0.1243 0.3899	−0.0803 0.5794	0.2248 0.1165	−0.1414 0.3274			
A6	0.2722 0.0559	−0.0476 0.7426	−0.1231 0.3944	−0.1562 0.2788	0.1249 0.3875		
A7	−0.0417 0.7739	−0.0292 0.8407	0.2387 0.0950	−0.0956 0.5088	0.1243 0.3899	0.2722 0.0559	
A8	−0.0417 0.7739	−0.0292 0.8407	0.2387 0.0950	−0.0956 0.5088	0.1243 0.3899	0.2722 0.0559	1.0000 <0.0001 *

^1^ Correlation coefficient; ^2^ Probability level; * Correlation data (also presented in italics) are significantly different at *p* < 0.05; Variable details: A1 = indicated ‘yes’ to familiar with good hygiene practices; A2 = indicated ‘yes’ to familiar with good storage practices; A3 = informally acquired good hygiene and storage practices; A4 = more than 5 years of slaughterhouse experience; A5 = indicated ‘no’ to prior knowledge/experience before joining the slaughterhouse; A6 = indicated ‘no’ to knowing any foodborne pathogen associated with beef; A7 = indicated ‘yes’ to knowing the importance of hand washing; A8 = indicated ‘yes’ on how to use the storage facilities in the slaughterhouse.

**Table 5:** Correlation coefficients obtained between highly responded perception aspects of good hygiene and storage practices of butchers.

Variable	B1	B2	B3	B4	B5	B6	B7
B2	0.5100 ^1^ 0.0002 * ^2^						
B3	0.6426 <0.0001 *	0.5743 <0.0001 *					
B4	0.2376 0.0967	0.3203 0.0234 *	0.1216 0.4004				
B5	0.4569 0.0009 *	0.4193 0.0024 *	0.1813 0.2077	0.4001 0.0040 *			
B6	0.5599 <0.0001 *	0.8430 <0.0001 *	0.7235 <0.0001 *	0.3469 0.0136 *	0.3451 0.0141 *		
B7	0.5345 <0.0001 *	0.8006 <0.0001 *	0.6889 <0.0001 *	0.3333 0.0180 *	0.3273 0.0203 *	0.9608 <0.0001 *	

^1^ Correlation coefficient; ^2^ Probability level; * Correlation data presented in italics are significantly different at *p* < 0.05; Variable details: B1 = indicated ‘very important’ for how do you perceive good hygiene/storage practice, in general; B2 = indicated ‘not so high’ for how do you perceive the hygiene level at the slaughterhouse facility; B3 = indicated ‘very high’ for how do you perceive the storage level at the slaughterhouse facility; B4 = indicated ‘yes’ to are there aspects of hygiene and storage at the slaughterhouse you believe require improvement; B5 = indicated ‘yes’ to do you believe the local, state, and federal governments have a role to play to enhance the slaughterhouse hygiene and storage facilities/practices; B6 = indicated ‘yes’ to do you believe the government regulation protects slaughterhouse good hygiene and storage practices; B7 = indicated ‘yes’ to do you believe the above (6) has helped to sustain slaughterhouse hygiene and storage facilities.

**Table 6:** Correlation coefficients obtained between highly responded knowledge and perception aspects of good hygiene and storage practices of butchers.

Variable	B1	B2	B3	B4	B5	B6	B7
A1	0.1000 ^1^	0.1961	0.0331	0.2722	0.1893	0.2125	0.2041
	0.4895 ^2^	0.1723	0.8196	0.0559	0.1879	0.1385	0.1551
A2	−0.0891	−0.1487	−0.1216	−0.0476	−0.0624	−0.1373	−0.1429
	0.5384	0.3028	0.4004	0.7426	0.6671	0.3419	0.3223
A3	0.0439	−0.0148	0.1846	0.0821	0.1746	0.0148	0.0000
	0.7623	0.9188	0.1995	0.5710	0.2252	0.9188	1.0000
A4	−0.2922	−0.4877	−0.3987	−0.1562	−0.2045	−0.4501	−0.3644
	0.0395 *	0.0003 *	0.0041 *	0.2788	0.1543	0.0010 *	0.0093 *
A5	−0.1418	−0.0225	−0.0987	0.1249	−0.1175	−0.1650	−0.1873
	0.3258	0.8768	0.4954	0.3875	0.4163	0.2523	0.1927
A6	−0.0594	0.1868	−0.0135	0.1111	0.0364	0.2135	0.2000
	0.6820	0.1939	0.9258	0.4424	0.8020	0.1366	0.1638
A7	*0.3273*	0.1961	0.2399	−0.0680	0.1893	0.2125	0.2041
	0.0203 *	0.1723	0.0934	0.6387	0.1879	0.1385	0.1551
A8	*0.3273*	0.1961	0.2399	−0.0680	0.1893	0.2125	0.2041
	0.0203 *	0.1723	0.0934	0.6387	0.1879	0.1385	0.1551

^1^ Correlation coefficient; ^2^ Probability level; *Correlation data (also presented in italics) are significantly different at *p* < 0.05; Variable details: A1 = indicated ‘yes’ to familiar with good hygiene practices; A2 = indicated ‘yes’ to familiar with good storage practices; A3 = informally acquired good hygiene and storage practices; A4 = more than 5 years of slaughterhouse experience; A5 = indicated ‘no’ to prior knowledge/experience before joining the slaughterhouse; A6 = indicated ‘no’ to knowing any foodborne pathogen associated with beef; A7 = indicated ‘yes’ to knowing the importance of hand washing; A8 = indicated ‘yes’ on how to use the storage facilities in the slaughterhouse; B1 = indicated ‘very important’ for how do you perceive good hygiene/storage practice, in general; B2 = indicated ‘not so high’ for how do you perceive the hygiene level at the slaughterhouse facility; B3 = indicated ‘very high’ to how do you perceive the storage level at the slaughterhouse facility; B4 = indicated ‘yes’ to are there aspects of hygiene and storage at the slaughterhouse you believe require improvement; B5 = indicated ‘yes’ to do you believe the local, state, and federal governments have a role to play to enhance the slaughterhouse hygiene and storage facilities/practices; B6 = indicated ‘yes’ to do you believe the government regulation protects slaughterhouse good hygiene and storage practices; B7 = indicated ‘yes’ to do you believe the above (6) has helped to sustain slaughterhouse hygiene and storage facilities.
